# Treating the symptom or treating the disease in neonatal seizures: a systematic review of the literature

**DOI:** 10.1186/s13052-021-01027-2

**Published:** 2021-04-07

**Authors:** Raffaele Falsaperla, Bruna Scalia, Andrea Giugno, Piero Pavone, Milena Motta, Martina Caccamo, Martino Ruggieri

**Affiliations:** 1 Neonatal Intensive Care Unit, A.O.U. San Marco-Policlinico, University of Catania, Via Carlo Azeglio Ciampi, 95121 Catania, Italy; 2 Post graduate programme in Pediatrics, Department of Clinical and Experimental Medicine, University of Catania, Catania, Italy; 3 Unit of Clinical Pediatrics, A.O.U. “Policlinico”, P.O. “G. Rodolico”, University of Catania, Catania, Italy; 4 Department of Clinical and Experimental Medicine Section of Pediatrics and Child Neuropsychiatry, A.O.U. San Marco- Policlinico, University of Catania, Catania, Italy

**Keywords:** Neonate, Seizure, Treatment, Outcome, Phenobarbital, Levetiracetam

## Abstract

**Aim:**

The existing treatment options for neonatal seizures have expanded over the last few decades, but no consensus has been reached regarding the optimal therapeutic protocols. We systematically reviewed the available literature examining neonatal seizure treatments to clarify which drugs are the most effective for the treatment of specific neurologic disorders in newborns.

**Method:**

We reviewed all available, published, literature, identified using PubMed (published between August 1949 and November 2020), that focused on the pharmacological treatment of electroencephalogram (EEG)-confirmed neonatal seizures.

**Results:**

Our search identified 427 articles, of which 67 were included in this review. Current knowledge allowed us to highlight the good clinical and electrographic responses of genetic early-onset epilepsies to sodium channel blockers and the overall good response to levetiracetam, whose administration has also been demonstrated to be safe in both full-term and preterm newborns.

**Interpretation:**

Our work contributes by confirming the limited availability of evidence that can be used to guide the use of anticonvulsants to treat newborns in clinical practice and examining the efficacy and potentially harmful side effects of currently available drugs when used to treat the developing newborn brain; therefore, our work might also serve as a clinical reference for future studies.

## Introduction

Pediatric patients develop seizures more frequently during the neonatal period than during any other age. The precise incidence of neonatal seizures can be difficult to define and depends on the population being studied and the criteria used for diagnosis. Neonatal seizures have been estimated to occur in up to3–5out of every 1000 births, and preterm newborns are estimated to develop seizures more frequently than full- term newborns, with an overall incidence of1 0–15 per1,000 preterm newborns, compared with 3–5 per 1000 full-term newborns and a prevalence of 22.2% among preterm newborns, compared with 0.5% among full term newborns [1, 2]. Moreover, because improved critical care has increased the survival rate following neonatal seizures, long-term neurological sequelae constitute a growing challenge for neonatologists. Greater than 50% of survivors, especially among preterm newborns, experience considerable disabilities across a range of developmental domains, with cerebral palsy and intellectual disability being the most frequently reported [2–4]. Recent studies have shown a 17.6% over- all incidence in epilepsy among children with a history of neonatal seizures [5].

Neonatal seizures are often misdiagnosed, resulting in both the under- and overestimation of clinically- diagnosed seizure occurrence, due to electro-clinical dissociation phenomena and because neonatal seizures are often highly focal, with very little spread to other brain regions [6–9]. Therefore, our review focused only on studies that described seizures confirmed by electroencephalography (EEG)- or amplitude-integrated EEG (aEEG).

In addition to making a correct diagnosis, making an early diagnosis of neonatal seizure is fundamental to the administration of proper treatment. Both animal and human studies have demonstrated that recurrent and prolonged seizures are harmful to the developing brain, emphasizing the importance of early seizure recognition and the availability of effective therapy options [10–14].

One of the major challenges facing clinicians who treat neonates with seizures is the lack of effective antiepileptic drugs (AEDs).

Advances on this front have occurred during the last few decades; the anticonvulsant properties of therapeutic hypothermia, for example, have been demonstrated by both preclinical and clinical data. However, a proper, specific, effective, and safe pharmacological treatment for neonatal seizures remains lacking.

Currently, the World Health Organization (WHO) recommends the use of phenobarbital and phenytoin as first-line treatment [15] options for neonatal seizures, despite the low-quality evidence available to support their efficacy and the number of studies highlighting their potential side effects, which include increasing neuronal apoptosis and, consequently, contributing to long-term neurological damage and adverse neurocognitive outcomes [16, 17].

Here, we systematically review the available evidence for the treatment of electrographic and electroclinical neonatal seizures caused by specific neurologic disorders in newborns and evaluate the efficacy of both first-line and add-on anticonvulsants. Data on the populations studied, the seizure etiologies, treatment protocols, and study strengths and limitations were collected [18, 19].

## Materials and methods

For this systematic review, we searched the PubMed database using search terms related to neonatal seizures (see below). The search period was from August 1949 to November 2020 (last update 30/11/2020). The only filters applied were publication in the English language and human studies.

A further search of ClinicalTrials.gov was conducted, and a list of ongoing clinical trials is provided.

### Search strategy

The systematic review was conducted following the general principles established by Preferred Reporting Items for Systematic Reviews and Meta-Analyses (PRISMA) and the Institute of Medicine Standards for Systematic Reviews [20, 21].

Given the lack of robust evidence [randomized clinical trials (RCTs)], we included observational investigations and case reports in our systematic review and focused on evaluating the strengths and methodological limitations of each included study.

The following search strategy was employed: (neonatal seizures treatment) OR (neonatal seizures AND treatment) OR (neonatal seizure antiepileptic drugs) OR (neonatal seizure AND antiepileptic drugs) OR (neonatal seizure phenobarbital) OR (neonatal seizure AND phenobarbital) OR (neonatal seizure phenytoin) OR (neonatal seizure AND phenytoin) OR (neonatal seizure lidocaine) OR (neonatal seizure AND lidocaine) OR (neonatal seizure levetiracetam) OR (neonatal seizure AND levetiracetam) OR (neonatal seizure carbamazepine) OR (neonatal seizure AND carbamazepine) OR (neonatal seizure topiramate) OR (neonatal seizure AND topiramate) OR (neonatal seizure midazolam) OR (neonatal seizure AND midazolam) OR (neonatal seizure valproic acid) OR (neonatal seizure AND valproic acid) OR (neonatal seizure lorazepam) OR (neonatal seizure AND lorazepam) OR (neonatal seizure lacosamide) OR (neonatal seizure AND lacosamide) OR (neonatal seizure lamotrigine) OR (neonatal seizure AND lamotrigine).

### Inclusion criteria

Seizures in full-term infants, only if they occurred within 30 days of birth;

Seizures in preterm infants, only if documented within the postmenstrual age (gestational age plus chronological age, in weeks) of 40 weeks;

Studies describing electro-clinical seizures;

Studies defining a precise etiology underlying seizure onset; and English language studies.

### Exclusion criteria

Studies describing a metabolic, reversible etiology for neonatal seizures, such as metabolic diseases, pyridoxin-dependent seizures, and electrolytic imbalance disturbances (hypoglycemia and hypocalcemia);

Articles that included EEG and semiology, but described patients who were not in the neonatal period; and Review articles, editorials, letters to the editor, and articles without individual descriptions of seizure semiology and/orEEG.

### Data collection and analysis

The reviewer screened the title and abstract of each study identified using the above-described search strategy. The same reviewer re-screened the full text of each study that was identified as potentially relevant. Studies meeting any of the pre-specified inclusion criteria were included.

### Methodological quality

Our systematic review was assessed using the “Assessing the Methodological Quality of Systematic Reviews 2” (AMSTAR 2) criteria. According to AMSTAR 2 score, “moderate quality review” result was obtained for this review [22].

### Description

**Fig. 1:**
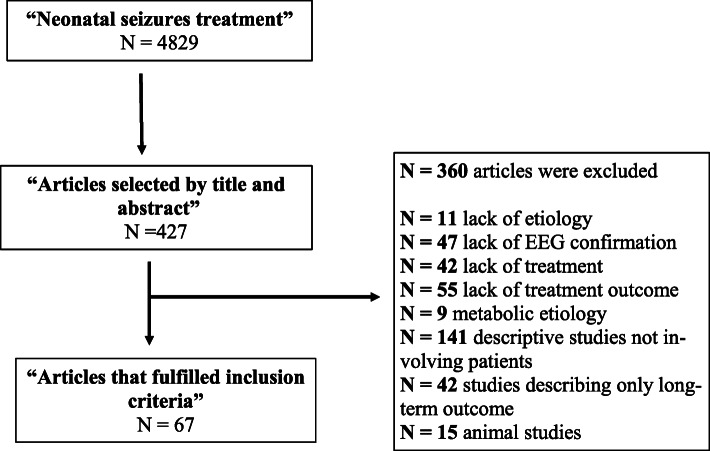
Search strategy and article selection

Our PUBMED search for “neonatal seizure treatment” identified 4.829 articles. After reviewing the titles and abstracts of these articles, the authors reviewed the full texts of 427 articles. A total of 67 of these fulfilled the criteria for inclusion in the review (Fig. 1).

Studies were grouped for type of study (RCTs, prospective, retrospective, case reports). In order to ease the access to the large amount of information provided by the literature, of each article we summarized in tables the number of patients included, the etiology beneath the seizures, first-line treatments, add-on therapies- when available-, and treatment outcomes. Data are listed in the tables below (Tables 1, 2, 3, 4).

**Table 1:** Full description of the sources: RCTs

RCTs	Population	Etiology	Treatment	Add-on therapy	Outcome
**Painter et al.** [23]	*N* = 59; term and preterm	*N* = 49 HIE *N* = 6 CNS infections *N* = 3 cryptogenic	N = 30 PHB *N* = 29 PHE Dose: N/A	*N* = 15 PHE as 2nd line AED *N* = 13 PHB as 2nd line AED	PHB’s efficacy: 43%. PHE’s efficacy: 45%. When combined, efficacy raised to 57–62%.
**Boylan et al.** [24]	*N* = 22; term and preterm.	*N* = 13 HIE *N* = 3 IVH *N* = 1 BFNE *N* = 2 IUGR *N* = 1 premature *N* = 1 myopathy *N* = 1 AVM	N = 22 PHB (20–40 mg/kg)	*N* = 3 MDZ *N* = 5 LID *N* = 3 CLZ	50% response to PHB. 2/5 responded to LID as 2nd line treatment. No response to CLZ and MDZ.
**Pressler et al.** [25]	*N* = 14; term.	HIE	BMT (0.05–03 mg/kg) + PHB (10 mg/kg)	*N* = 8 MDZ *N* = 5 PHE *N* = 2 LID *N* = 4 PHB	5/14 had seizure cessation on BMT + PHB. 3/14 had hearing loss.
**Falsaperla et al.** [26]	*N* = 30; term.	*N* = 23 HIE *N* = 3 stroke *N* = 4 CNS infection	PHB (20 mg/kg load, maintenance 5 mg/kg) OR LEV (load dose 20 mg/kg, maintenance 20–40 mg/kg)	/	LEV’s administration related to a significantly positive HNNE score. There was no significant improvement in the HNNE score after one month in the neonates treated with PHB.

*N* number of patients; *PHB* phenobarbital; *PHE* phenytoin; *CNS* central nervous system; *AED* anti-epileptic drug; *N/A* not available; *HIE* hypoxic ischemic encephalopathy; *IVH* intra-ventricular hemorrhage; *BFNE* benign familial neonatal epilepsy; *IUGR* intra-uterine growth restriction; *AVM* arterio-venous malformation; *MDZ* midazolam; *LID* lidocaine; *BMT* bumetanide; *LEV* levetiracetam. *EEG* electroencephalography, *HNNE* Hammersmith Neonatal Neurological Examination; *c.i.* continuous infusion; *HHNE* Hammersmith Neonatal Neurological Examination

**Table 2:** Full description of the sources: prospective studies

Prospective studies	Population	Etiology	Treatment	Add-on therapy	Outcome
**Ramantani et al.** [27]	*N* = 38; term and preterm.	*N* = 9 HIE *N* = 16 IVH *N* = 13 sepsis	*N* = 38 LEV (10–30 mg/kg)	*N* = 11 PHB as 2nd line	LEV’s efficacy: 79%; 27/30 (90%) remained seizure free at 4 weeks.
**Sharpe et al.** [28]	*N* = 18; term.	*N* = 8 HIE *N* = 2 IVH *N* = 1 birth trauma *N* = 2 stroke *N* = 1 brain malformation *N* = 4 N/A	PHB (20 mg/kg)	LEV (20-40 mg/kg)	LEV’s efficacy: 42%. 5 of the responders were among the 12 subjects who received the higher dose of LEV.
**Furwentsches et al.** [29]	*N* = 6; term and preterm	*N* = 1 HIE *N* = 1 IVH *N* = 3 brain malformation *N* = 1 N/A	*N* = 4 oral LEV (10–50 mg/kg) *N* = 2 PHB (10 mg/kg)	*N* = 2 LEV	All 6 patients treated with oral LEV became seizure free within 6 days. After 3 months, five out of six patients were seizure free under monotherapy with LEV.
**Falsaperla et al.** [30]	*N* = 16; late preterm and term.	*N* = 5 MAS *N* = 8 RDS *N* = 3 APD	LEV (10–40 mg/kg)	/	LEV’s efficacy: 100%.Seizure freedom reached from 24 h to 15 days. EEGs at three months resulted normal.
**Boylan et al.** [31]	*N* = 17	*N* = 7 HIE *N* = 1 RDS *N* = 1 IUGR *N* = 3 sepsis *N* = 3 prematurity *N* = 1 NAS *N* = 1 kernicterus	*N* = 15 PHB (20–30 mg/kg)	*N* = 2 PHE (20 mg/kg)	Full response to PHB in 6/17 (35%). Partial response to PHB in 6/17 (35%). No response in 2/17 (12%).
**Low et al.** [32]	*N* = 19 term or near term	*N* = 10 HIE *N* = 6 stroke *N* = 1 CNS infection *N* = 1 BFNE *N* = 1 N/A	PHB 10–40 mg/kg i.v.	PHE or MDZ as 2nd line (dose: N/A)	PHB abolished seizurs in 13/19 patients within 1 h. Only 3 patients showed permanent reduction. Loading dose of 20 mg/kg was more effective than 10 mg/kg.
**Van der Broek et al.** [33]	*N* = 53 term newborns	HIE	PHB 20–40 mg/kg	MDZ c.i. 0.05–0.1 mg/kg/h	The observed responsiveness of MDZ add-on therapy after PHB monotherapy was low (23%) compared to PHB monotherapy’s effectiveness (66%).
**Hellstrom- Westas et al.** [34]	*N* = 24 both term and preterm	*N* = 15 HIE *N* = 6 IVH *N* = 2 HIE *N* = 1 hypoglicemia	*N* = 24 PHB **(**10–15 mg/kg)	*N* = 21 DZP as 2nd line (0.5–2 mg/kg) *N* = 24 LID as 3rd line (1.6–2.2 mg/kg)	LID’s introduction conducted to seizure cessation in 15/24 patients. 2 of them developed bradycardia and acidosis.
**Maytal et al.** [35]	*N* = 7	N = 4 HIE *N* = 2 IVH *N* = 1 CNS infections	*N* = 7 PHB (20–40 mg/kg)	*N* = 7 LRZ (0.05 mg/kg i.v.)	After PHB’s failure, 6/7 patients had complete cessation of seizures within 3 min. 4/6 remained seizure-free on follow-up. No side-effects were reported.
**Glass et al.** [36]	*N* = 92	*N* = 30 HIE *N* = 25 IVH	*N* = N/A PHB (20 mg/kg) *N* = N/A LEV (dose: N/A) *N* = N/A PHE (dose: N/A)	N/A	64% had seizures that persisted after loading doses of PHB, 58% after LEV and 100% after PHE. .
**Glass et al.** [37]	*N* = 543 term and preterm	*N* = 284 HIE *N* = 142 stroke *N* = 108 IVH	*N* = 508 PHB *N* = 21 LEV *N* = 5 PHE (doses: N/A)	N/A	354/543 (66%) neonates had incomplete response to the initial loading dose of AED. Incomplete response was similar for PHB (66%), LEV (67%), PHE (80%).

*N* number of patients; *PHB* phenobarbital; *PHE* phenytoin; *CNS* central nervous system; *AED* anti-epileptic drug; *N/A* not available; *HIE* hypoxic ischemic encephalopathy; *IVH* intra-ventricular hemorrhage; *BFNE* benign familial neonatal epilepsy; *IUGR* intra-uterine growth restriction; *MDZ* midazolam; *LID* lidocaine; *BMT* bumetanide; *MAS* meconium aspiration syndrome; *RDS* respiratory distress syndrome; *LEV* levetiracetam. *EEG* electroencephalography; *LRZ* lorazepam; *DZP* diazepam; *CFM* cerebral function monitoring; *NAS* neonatal abstinence syndrome; *APD* acute placental detachment

**Table 3:** Full description of the sources: retrospective studies

Retrospective studies	Population	Etiology	Treatment	Add-on therapy	Outcome
**Abend et al.** [38]	*N* = 23 late preterm and term	*N* = 8 HIE *N* = 4 genetic *N* = 3 malformative *N* = 3 infections *N* = 2 cryptogenic *N* = 2 stroke *N* = 1 tumor	*N* = 18 PHB (dose: N/A) *N* = 1 PHE (dose: N/A) *N* = 4 LEV (10–20 mg/kg)	*N* = 13 LEV as 2nd line (dose: N/A) *N* = 5 LEV as 3rd line	Seizure cessation in 7/23 (30%); seizure reduction (> 50%) in 1/23.
**Khan et al.** [39]	*N* = 22 term	*N* = 12 HIE *N* = 2 IVH *N* = 2 CNS infections *N* = 6 various	*N* = 16 PHB (dose: N/A) *N* = 3 LEV (dose: N/A)	*N* = 19 received LEV as 2nd (*N* = 16), 3rd (N = 2) or 4th (*N* = 1) line	7 of 22 patients (32%) achieved complete cessation of seizures after administration of the loading dose, 14 (64%) achieved cessation of seizures by 24 h, 19 (86%) by 48 h, and all 22 (100%) by 72 h
**Khan et al.** [40]	*N* = 12 preterm	*N* = 5 HIE *N* = 3 IVH *N* = 3 N/A *N* = 1 HSV encephalitis	*N* = 9 PHB (dose: N/A) *N* = 3 LID (dose: N/A)	*N* = 12 LEV (dose: N/A)	4 patients (36%) reached seizure cessation after the loading dose, 9 (82%) by 24 h, 10 (91%) by 48 h, and 10 subjects (91%) by 72 h.
**Rakshabhuva-** **nkar et al.** [41]	*N* = 8 term and preterm	*N* = 5 HIE *N* = 2 IVH *N* = 1 N/A	*N* = 8 PHB (dose: N/A) *N* = N/A PHE (dose: N/A)	*N* = 8 LEV (10 mg/kg)	LEV’s effectiveness in 6/8 patients.
**Lo Yee Yau et al.** [42]	*N* = 12 preterm and term	*N* = 6 HIE *N* = 3 CNS infections *N* = 1 hypoglicemia *N* = 2 metabolic	*N* = 12 PHB (dose: N/A)	N = 8 MDZ as 2nd line AED and LEV as 3rd line. *N* = 4 were given LEV as 2nd line AED.	LEV’s efficacy: 75% of patients treated. No side effects reported.
**Maljevic et al.** [43]	*N* = 10	KCNQ 3 mutations	*N* = 1 PYR *N* = 2 LEV *N* = 1 OXC	N = 1 LEV (65 mg/kg) *N* = 1 OXC (20 mg/kg)	1 was seizure free after one dose of LEV. 1 was seizure free after day 20 on LEV. 1 was seizure free on OXC.
**Shin et al.** [44]	*N* = 18 term and preterm	*N* = 12 HIE *N* = 1 CNS *N* = 1 IVH *N* = 4 malformative	*N* = 18 PHB or PHE (dose: N/A)	*N* = 18 LEV after PHB failure (N = 1 only LEV *N* = 11 LEV + PHB *N* = 6 PHE + LEV)	94% of patients had seizure cessation within the first week from LEV’s introduction, and 89% remained seizure-free under LEV monotherapy at 1 month.
**Han et al.** [45]	*N* = 37 preterm	*N* = 15 HIE *N* = 6 GMH *N* = 14 IVH *N* = 1 malformative *N* = 1 meningitis	LEV (40–60 mg/kg)	N = N/A PHB (20 mg/kg) *N*=N/A PHE, MDZ, TPM, VPA as 3rd line.	Seizure cessation in 21 patients (57%) with LEV alone. Seizure cessation in 9 infants (24%) after LEV + PHB. 7 required third-line AED.
**Venkatesan et al.** [46]	*N* = 32 term	HIE	*N* = 23 PHB *N* = 2 LEV *N* = 2 MDZ (doses: N/A)	N = 23 LEV as 2nd line *N* = 2 LEV as 3rd line after PHB and MDZ failure	84% of the patients treated with LEV achieved seizure cessation within 72 h.
**Rao et al.** [47]	*N* = 44 term	HIE	*N* = 23 PHB *N* = 2 LEV	*N* = 10 LEV as 2nd line	50% of patients treated with levetiracetam became seizure-free after 40 h, and 100% achieved seizure freedom between 100 and 120 h.
**Van der Broek et al.** [48]	*N* = 31 term	HIE	*N* = 31 PHB (20 mg/kg)	/	PHB’s efficacy: 66%.
**Boylan et al.** [49]	*N* = 14 term and preterm	*N* = 4 HIE *N* = 3 IVH *N* = 3 metabolic *N* = 1 meningitis *N* = 3 mild asphyxia	*N* = 14 PHB (20–40 mg/kg)	*N* = 4 CLZ *N*=N/A PHE (doses: N/A)	PHB was only effective in 29% of patients, those with normal background EEGs or mild to moderate background abnormalities and low seizure burden.
**Spagnoli et al.** [50]	*N* = 91 term and preterm	*N* = 45 HIE *N* = 21 IVH *N* = 4 malformative *N* = 12 metabolic disorders *N* = 4 CNS infection *N* = 1 genetic *N* = 4 N/A	*N* = 91 PHB (20 mg/kg)	*N* = N/A PHE 20 mg/kg as 2nd line *N* = N/A MDZ 0.15 mg/kg as 3rd line	PHB was effective alone in 62.6% of patients.
**Hakeem et al.** [51]	*N* = 11 term and preterm	*N* = 6 HIE *N* = 3 IVH *N* = 1 CNS *N* = 1 N/A	*N* = 2 oral cloral hydrate (30 mg/kg) *N* = 7 PHB (20 mg/kg) *N* = 2 DZP (1 to 2 mg iv bolus)	*N* = 1 CLZ c.i. (10 mcg/kg/hr)	6/7 responded to PHB but 4/7 later relapsed.
**Weeke et al.** [52]	*N* = 413 (*N* = 319 term, *N* = 94 preterm)	*N* = 228 HIE *N* = 45 HIE *N* = 32 PAIS *N* = 40 CNS infections *N* = 100 others	*N* = 413 PHB (dose: 20 mg/kg)	*N* = 186 LID as 2nd line *N* = 172 LID as 3rd line	In term infants, a response to LID was seen in 72.5–80%, with cessation of seizures and no need for rescue AED in 21.4–67.6%. Lower response rate in preterm (55.5–58.2% with cessation of seizures and no other AED in only 16.4–40.7%).
**Lundqvist et al.** [53]	*N* = 30 term	*N* = 18 HIE *N* = 4 meningitis *N* = 6 PAIS *N* = 1 hypoglicemia *N* = 1 uncertain	N = 17 DZP (dose: N/A) *N* = 8 MDZ (dose: N/A) *N* = 5 DZP + MDZ (dose: N/A)	*N* = 30 LID	LID’s efficacy: 65%.
**Van der Broek et al.** [54]	*N* = 22 term	HIE	*N* = 22 PHB (dose: N/A)	*N* = 22 MDZ (dose: N/A) *N* = 22 LID (dose: 2 mg/kg, followed by 4 mg/kg/h c.i.)	20/22 (90%) newborns responded to LID. No cardiac arrythmias were reported (91%)
**Jennekens et al.** [55]	*N* = 11 term	*N* = 11 stroke	*N* = 11 PHB (20 mg/kg)	*N* = 8 MDZ as 2nd line *N* = 9 LID as 3rd line	In term newborns with PAIS, MDZ and LID induce a shift from lower to higher frequency electrocortical activity. Compared to LID, MDZ reduced more pronouncedly the total EEG power.
**Shany et al.** [56]	*N* = 30 term	*N* = 30 HIE	*N* = 30 DZP or PHB	*N* = 22 LID as 2nd line *N* = 8 MDZ as 2nd line	77% response rate to LID.
**McDermott et al.** [57]	*N* = 10 term	HIE	*N* = 5 LRZ *N* = 4 PHB or PHE (20 mg/kg) *N* = 1 PHB (20 mg/kg)	*N* = 5 LRZ as 2nd line	Administration of a single dose of LRZ stopped seizures in all neonates. 4 neonates receiving simultaneously PHB and/or PHE had no further seizures. 6 had seizure recurrence.
**Castro Conde et al.** [58]	*N* = 13 term and preterm	*N* = 7 HIE*N* = 3 stroke *N* = 2 IVH *N* = 1 N/A	*N* = 32 PHB (20 mg/kg tritated up to 40 mg/kg) followed by PHE as 2nd line AED (20 mg/kg)	*N* = 13 MDZ as 2nd (9/13) or 3rd (4/13) line AED in non-responders.	Ten of 13 neonates with SE treated with midazolam were electrically controlled in the first hour of treatment.
**Vilan et al.** [59]	*N* = 9 term	KCNQ2 mutations	*N* = 9 PHB	*N* = 8 PYR, *N* = 6 LID, *N* = 6 MDZ, *N* = 3 CZP, *N* = 3 LEV, *N* = 2 PHE, *N* = 2 VPA, *N* = 2 CBZ, *N* = 1 TPM	PH and PYR (used in 8/9 patients) were ineffective. 2 patients were SF during LID infusion and were later switched to oral PHT or oral CBZ.
**Montesclaros Hortigüela et al.** [60]	*N* = 13 G.A. = N/A	KCNQ2 mutations	*N* = 10 PHB *N* = 2 LEV *N* = 1 MDZ (doses: N/A)	Several AED were administered as 2nd line: CBZ, LEV, MDZ, VPA, PHE, TPM, VGB, LID, OXC, PYR	5/9 were seizure free but with severe impairment in psychomotor development in treatment with CBZ (*n* = 2), VPA + CBZ + LCM (*n* = 1), PHB + VPA + OXC (*n* = 1), OXC + TPM (*n* = 1).
**Pisano et al.** [61]	*N* = 15 term	KCNQ2 mutations	Multiple AEDs (including PHB as first-line AED, VPA, steroids) were tried unsuccessfully	*N* = N/A CBZ (20 mg/kg/day) *N* = N/A PHE (dose widely ranging)	53% of the patients were seizure-free on CBZ; 33% responded to PHE; the remaining 47% of the patients responded to TPM and LEV.
**Sands et al.** [62]	*N* = 19 term	SLC13A5 mutations (KCNQ2 gene)	*N* = 13 PHB (dose: N/A) *N* = 4 CBZ (10 mg/kg)	*N* = 15 CBZ (10 mg/kg)	CBZ’s efficacy: 89%.
**Singh et al.** [63]	*N* = 10 term	*N* = 8 HIE *N* = 2 unknown	*N* = 10 CBZ (dose: 10 mg/kg)	*N* = 2 DZP	Seizure control in 80% of patients on CBZ; 2 patients needed DZP as 2nd line.
**Glass et al.** [64]	N = 6 term	HIE	N = 5 PHB (30–60 mg/kg)	N = 5 TPM (10 mg/kg)	3/5 patients treated with TPM had seizure reduction or cessation. One adjunctive patient achieved seizure freedom on TPM at 6 months.

*N* number of patients; *PHB* phenobarbital; *PHE* phenytoin; *CNS* central nervous system; *AED* anti-epileptic drug; *N/A* not available; *HIE* hypoxic ischemic encephalopathy; *IVH* intra-ventricular hemorrhage; *BFNE* benign familial neonatal epilepsy; *IUGR* intra-uterine growth restriction; *MDZ* midazolam; *LID* lidocaine; *BMT* bumetanide; *MAS* meconium aspiration syndrome; *RDS* respiratory distress syndrome; *LEV* levetiracetam. *EEG* electroencephalography; *LRZ* lorazepam; *DZP* diazepam; *CFM* cerebral function monitoring; *NAS* neonatal abstinence syndrome; *VPA* valproic acid; *CBZ* carbamazepine; *TPM* topiramate; *G.A.* gestational age; *SE* status epilepticus; *HSV* herpes simplex virus; *PYR* pyridoxine; *OXC* oxcarbazepine; *GMH* germinal matrix hemorrhage; *TPM* topiramate; *CLZ* clonazepam; *DZP* diazepam; *LRZ* lorazepam; *PAIS* perinatal arterial ischemic stroke; *SF* seizure- free

**Table 4:** Full description of the sources: case reports

	Population	Etiology	Treatment	Add-on therapy	Outcome
**Shoemaker et al.** [65]	*N* = 3 term and preterm	*N* = 1 PAIS *N* = 2 PHVD	*N* = 2 PHB and PHE (dose: N/A) *N* = 1 PHE and OXC (dose: N/A)	*N* = 3 LEV as 3rd line (dose: N/A)	LEV’s administration resulted in seizure control in all three patients.
**Tanriverdi et al.** [66]	*N* = 1 term	SWS	PHB (20 mg/kg)	PHE as 2nd line (20 mg/kg) LEV as 3rd line (20 mg/kg)	Seizure control was achieved after LEV intravenous infusion.
**Hmaimess et al.** [67]	*N* = 1 Term	KCNT1 mutation	PHB (dose: N/A)	PHT, LTG, CLZ LEV (10–30 mg/kg)	LEV’s introduction resulted in dramatic decrease in seizure activity by the eighth day of treatment.
**Ledet et al.** [68]	*N* = 1 term	LLA	PHB (20 mg/kg)	LEV (40 mg/kg)	The patient was seizure-free on PHB and maintained seizure freedom on LEV that minimally interfered with her other ongoing treatments.
**Li Jiang et al.** [69]	*N* = 9 term	STXBP1mutations	PHB (dose: N/A)	5 patients did not respond and were tried on several AEDs (TPM, NZP, LEV, VPA, VIT B6, PDN, ACTH, KD)	44.4% of cases (4/9) in our study showed apparent responses to LEV.
**Dilena et al.** [70]	*N* = 1 term	SCN2A	PHB (dose: N/A)	LEV + PYR as 2nd line PHE as 3rd line (12–18 mg/kg/day)	Seizure freedom was reached on PHE, first, and maintained on oral CBZ.
**Bonhorst et al.** [71]	*N* = 1 term	KCNQ2	PHB (20 mg/kg) + VIT B6 (30 mg/kg/d)	MDZ c.i. as 2nd line (0.25 mg/kg/h) TPM as 3rd line (2 mg/kg/day) LID (6 mg/kg), then switched to PHE and, later CBZ (dose: N/A)	Seizure freedom was reached on LID; the patient developed methemoglobinemia as side-effect and seizure freedom was maintained with PHE, first, and oral CBZ, later.
**Numis et al.** [72]	*N* = 3 term and late preterm	KCNQ2 encephalopathy	PHB (dose: N/A)	LEV, TPM, VGB, CLZ, KD failed. CBZ was initiated at 3, 4 and 13 months (dose: N/A)	2/3 patients responded to CBZ and were seizure free at 30 months though developed severe psychomotor delay, quadriplegia, axial hypotonia with appendicular hypertonia, and a tendency to opisthotonos.
**Spagnoli et al.** [73]	*N* = 1 term	EIMFS due to KCNQ2 mutations	PHB (dose N/A)	PYR, LEV, PHE, MDZ, TPM, NTZ CBZ (dose: N/A)	After multiple AEDs failure, seizure ceased after 3 weeks from CBZ’s introduction. Patient was seizure free at nine months.
**Blumkin et al.** [74]	*N* = 1 Term	KNCQ2	PHB (dose: N/A)	TPM, LEV, VPA, LTG, PYR, folinic acid CBZ (50 mg/kg)	Seizure control was initially achieved with TPM. Seizures reoccurred after 3 weeks and did not respond to several AEDs until CBZ.
**Buttle et al.** [75]	*N* = 1 term	KCNQ2	PHB (dose: N/A)	LEV, LRZ, CLZ, PYR LID (2–4 mg/kg/h) CBZ (40 mg/kg)	After several AEDs failed, seizure freedom was reached on LID and maintained at a 13 months follow-up on oral CBZ.
**Soldovieri et al.** [76]	*N* = 1 term	KCNQ2 mutation (Kv7.2 subunit)	PHB (dose: N/A)	PYR, LEV, PHE, TPM, OXC	Partial response to an association of PHB, PHE, TPM. At 5 months he was switched to OXC and maintained seizure freedom until 14 months. The patient developed severe DD.
**McNally et al.** [77]	*N* = 1 term	SCN8A	PHB (20 mg/kg) + LEV (20–60 mg/kg)	OXC (up to 80 mg/kg) + PHE (20 mg/kg) + LTG (2 mg/kg/day)	The association of three sodium channel blockers (OXC + PHE + LTG) reduced seizures’ frequency.
**Okumura et al.** [78]	*N* = 1 term	2q21-q31deletion (SCN1A cluster)	PHB (dose: 20 mg/kg)	LEV as 2nd line (40 mg/kg) VPA as 3rd line (50 mg/kg)	PHB and LEV failed to control seizures; VPA reduced seizures’ frequency.
**Riesgo et al.** [79]	*N* = 3 preterm and term	*N* = 1 NAS *N* = 1 fetal distress *N* = 1 PVL	PHB (dose: N/A)	TPM (0.5–8 mg/kg/d) after several other AEDs failed (PHB, PHE, CLZ, VPA, MDZ)	Seizure cessation in all three after TPM’s administration.
**Sirsi et al.** [80]	*N* = 3 term	*N* = 1 HIE *N* = 1 meningitis *N* = 1 EIEE	PHB (dose: N/A)	PHE as 2nd line (dose: N/A) MDZ as 3rd line (up to 0.2 mcg/kg/h)	Seizure control within 6–72 h after MDZ’s introduction. One patient developed hypotension, that responded to inotropic support.
**Steinberg et al.** [81]	*N* = 2 preterm	*N* = 1 IVHN = 1 PVL	PHB (20 mg/kg)	PHE as 2nd line (20 mg/kg) Rectal VPA as 3rd line (20–30 mg/kg)	Seizure control was achieved and maintained on a 12 months follow-up on VPA.
**Tarocco et al.** [82]	*N* = 1 late preterm	Pierre-Robin, polymicrogyria, lissencephaly	PHB (dose: N/A)	PHE, MDZ, LEV, PPF KTM (2 mg/kg + c.i. of 10 mcg/kg/min)	Immediate complete clinical and electrographic response was obtained after KTM introduction; after 15 days SE relapsed and the patient died.
**Baxter et al.** [83]	*N* = 3 term	*N* = 2 EIEE *N* = 1 Aicardi-Goutieres	PHB (dose: N/A)	PYR, CLZ, VPA VGB (40 mg/kg/d)	2/3 patients showed full response to VGB.
**Wolf et al.** [84]	*N* = 1 term	Incontinentia pigmenti	PHB (35 mg/kg)	LRZ as 2nd line (0.2 mg/kg) PHE as 3rd line (20 mg/kg) Dexamethasone (0.25 mg/kg/d)	Rapid improvement and clinical seizures termination followed the initiation of CCS therapy.
**Shevell et al.** [85]	*N* = 1 term	BFNE	PHB (10 mg/kg)	/	Patient presented no more seizures, was discharged home on oral PHB, suspended at five months of life
**Lee et al.** [86]	*N* = 1 term	KCNQ2	PHB (6 mg/kg/day)	PHE as 2nd line (8 mg/kg/day) VGB (50 mg/kg/day)	VGB reduced seizures; Once treatment with Vigabatrin was administered seizures reduced to one per day until day 24 of post-natal life, time at which the last seizure was recorded.
**Sato et al.** [87]	N = 2 late preterm and term	HIE	PHB (10 mg/kg)	/	Both patients temporarily controlled seizures on PHB. One relapsed and developed severe DD.
**Sillanpää et al.** [88]	*N* = 1 term	Feeding epilepsy	PHB (60 mg/day) + chlorpromazine (9 mg/day)	The patient was seizure-free since day 14 of PHB.	Only few cases of neonatal feeding seizures are described. In this case the patient was seizure-free on PHB, after a six days combination-therapy with chlorpromazine.
**Tramonte et al.** [89]	*N* = 1 term	Temporal lobe hemorrhage	PHB (dose: N/A)	/	After PHB’s administration no more autonomic seizures (apnea, desaturations) were noticed.

*N* number of patients; *PHB* phenobarbital; *PHE* phenytoin; *CNS* central nervous system; *AED* anti-epileptic drug; *N/A* not available; *HIE* hypoxic ischemic encephalopathy; *IVH* intra-ventricular hemorrhage; *BFNE* benign familial neonatal epilepsy; *IUGR* intra-uterine growth restriction; *MDZ* midazolam; *LID* lidocaine; *LEV* levetiracetam. *EEG* electroencephalography; *LRZ* lorazepam; *VPA* valproic acid; *CBZ* carbamazepine; *TPM* topiramate; *PYR* pyridoxine; *OXC* oxcarbazepine; *TPM* topiramate; *CLZ* clonazepam; *DZP* diazepam; *LRZ* lorazepam; *PAIS* perinatal arterial ischemic stroke; *VGB* vigabatrin; *LLA* acute lymphoblastic leukemia; *PHVD* post-hemorrhagic ventricular dilatation; *SWS* Sturge Weber syndrome; *NZP* nitrazepam; *ACTH* adrenocorticotropic hormone; *PDN* prednisone; *LTG* lamotrigine; *PVL* periventricular leukomalacia; *EIEE* early infantile epileptic encephalopathy; *PPF* propofol; *KTM* ketamine; *EIMFS* early infantile migrating focal seizures; *DD* developmental delay

### Future directions

A further search on ClinicalTrials.gov for the terms “neonatal seizures” and “neonatal seizures treatment” led to the identification of 5 ongoing clinical trials.

A multicenter, open-label, single-arm study to evaluate the pharmacokinetics, efficacy, and safety of brivaracetam in a cohort of 42 full-term or near-term neonates with repeated electroencephalographic seizures (NCT03325439) is currently recruiting and is estimated to be completed in December 2021.

LEVNEONAT-1 (NCT02229123), is an open-label study evaluating the efficacy and optimal dose of intravenous levetiracetam as a first-line treatment in full-term or near-term (36–43-week gestational age) newborns with HIE. Patients will be treated with 1 loading dose of 30, 40, 50, or 60 mg/kg and 8 quarter-loading maintenance doses for a 3-day treatment. A dose with toxicity not exceeding 10% and an efficacy greater than 60% will be considered the optimal dose. Efficacy is defined by the authors as a seizure burden reduction of 80% after the initial loading dose. The minimal sample expected is 50 participants, with a minimum of 24 patients, although fewer will be used in case of high toxicity.

Another Phase IIb randomized, blinded, controlled study (NCT01720667), which involves 6 different centers in the United States, is currently evaluating the efficacy of levetiracetam for terminating seizures when given as a first-line anticonvulsant in full-term newborns. A large cohort of 280 patients has been enrolled. The efficacy of intravenous levetiracetam (40 to 60 mg/kg intravenously, followed by a 30 mg/kg/day maintenance dose) will be compared with that for phenobarbital (20 to 40 mg/kg, followed by 1.5 mg/kg every 8 h).

A multicenter randomized, blinded, controlled, study examining the efficacy of oral levetiracetam as a first-line anticonvulsant in China (NCT02550028) is planning to enroll 100 full-term newborns, with EEG-confirmed seizures, and randomly assign them into either an interventional group treated with intravenous levetiracetam (50 mg/kg, followed by 30 mg/kg/day) or a control group treated with phenobarbital (20 mg/kg), with the aim of describing the efficacy of levetiracetam continuous EEG monitoring.

Recently, a randomized, double-blind, parallel-group, phase III study (NCT03602118), with the aim of evaluating the efficacy of phenobarbital sodium injections in participants who have suffered from clinical seizures, has been reported. Because neonatal seizures can have long-term side effects, including death, placebo-controlled studies are not appropriate for this population. The study is designed to demonstrate the effectiveness of phenobarbital for the prevention of subsequent seizures and to demonstrate improved efficacy when used at a higher dose (40 mg/kg) compared with a lower dose (20 mg/kg). Study participants who experience electrographic or electroclinical seizures that last for 10 s or longer will be randomized, in a 1:1 fashion, between the 2 treatment arms to receive either a 20 mg/kg or 40 mg/kg loading dose of phenobarbital sodium. Participants in the 20 mg/kg treatment arm in whom seizure activity does not resolve after the first dose will receive phenobarbital in 10 mg/kg increments (each hour) until seizure activity resolves, up to a maximum dose of 40 mg/kg. If seizure activity still does not resolve, participants will be given a second-line anticonvulsant. Participants in the 40 mg/kg group in whom seizures do not resolve after the initial loading dose will be given a second-line anticonvulsant. The second line treatment will be determined by the attending physician based, on the patient’s clinical history and the seizure’s features.

## Results

In the 67 articles included in this review (4 RCT, 11 prospective studies, 27 retrospective studies and 25 case reports), HIE, stroke and genetic channelopathies were the most frequent etiologies of seizures. Despite the number of patients described in this review, performing statistical analyses of the data and providing precise descriptions for how the considered anticonvulsants work was challenging. In an attempt to standardize the results, we grouped all neonates with specific seizure etiologies as though they belonged to a single study. This decision was made because most studies analyzed small populations, which were too small for statistical analysis; however, this method provided us with the opportunity to analyze the whole dataset as 1 large cohort of patients. Overall, 556 patients whit HIE, 45 patients whit stroke and 76 patients whit genetic channelopathies were considered.

### Limitations

The primary limitation when interpreting study results was that most studies analyzed heterogeneous populations, including both full-term and preterm newborns, with seizures caused by a variety of etiologies, which prevented the assessment of relationships between seizure etiology and treatment efficacy, in most cases. In addition, no consensus regarding the definition of treatment efficacy is available in the literature; therefore, each study relied on a unique definition of efficacy, which ranged from a “seizure reduction of more than 80% within an hour from the drug administration,” to “a global seizure burden reduction during the response period on EEG-monitoring”, to “greater than 30 % seizure reduction compared to another medication”. The authors more often referred to an overall efficacy, without specifying the time from drug administration to seizure burden reduction as assessed by EEG monitoring.

Another limitation was that most of the available studies described combination treatments, resulting in the possible misinterpretation of each drug’s specific effectiveness. In addition, as previously mentioned, we were unable to perform a meta-analysis because many studies included examined small, heterogeneous patient populations.

### Hypoxic-ischemic encephalopathy

Among the studies reported in our review, 11 described homogeneous populations of newborns with HIE. Overall, 556 newborns with HIE were described (Table 5).

**Table 5:** Treatment outcomes in patients with hypoxic-ischemic encephalopathy

	N of patients treated	N of patients treated as 1st-line	Efficacy as 1st-line AED; N (%)	N of patients treated as 2nd-line	Efficacy as 2nd-line; N (%)	N of patients treated as 3rd-line	Efficacy as 3rd-line; N (%)	Overall efficacy	Side-effects
**Phenobarbital**	76	76	49 (65%)	/	/	/	/	65%	None reported
**Lorazepam**	10	5	2 (20%)	5	2 (20%)		/	4 (40%)	Liver enzyme elevation in 1 patient
**Midazolam**	333	/	/	226	14–50%	107	57.5%	36%	Hypotension in 39 patients (12%)
**Lidocaine**	317	/	/	208	94 (45%)	125	91 (73.4%)	59%	None reported
**Levetiracetam**	76	22	Seizure freedom in 11 (50%) patients after 40 h, in 22 (100%) after 5 days	54	49 (92%)	/	/	75%	None reported
**Bumetanide**	14	14	7 (50%)	/	/	/	/	N/A	Ototoxicity

In a cohort of 76 asphyxiated newborns treated with phenobarbital, as a first-line anticonvulsant, efficacy was reported in 65% of cases, which is in line with previously available reports in the literature [33, 47, 48, 50, 87].

Among benzodiazepines, lorazepam was used as a first- or second-line anticonvulsant in a small cohort of 10 asphyxiated patients, with an overall 40% response; however, the 52 patients who did respond, were being treated simultaneously with phenobarbital, making it impossible to determine whether either drug individually or the combination was actually effective.

When used as a second-line anticonvulsant in 226 newborns after phenobarbital failure, midazolam was reported to be effective in 32% (14–50%) of the patients treated [48, 56]. Reports from 107 newborns treated with Midazolam as a third-line treatment described an overall efficacy of 57.5% [50]. These results appeared to be more promising during the first minutes after the initial administration (more than 80% seizure reduction within minutes after the first dose in 53 patients), but only half of the patients maintained seizure reduction over a 24-h period of observation. Serious hypotensive episodes were reported in 39 (12%) patients treated.

A total of 317 patients were treated with lidocaine as a second- or third-line anticonvulsant, with a reported overall response to treatment of 45%, when used as second-line treatment, [52, 56] and of 73.4%, when used as a third-line anticonvulsant [52, 54]. Unfortunately, a closer look at the patient’s features and hypoxia severity scores revealed that good responses were achieved in less severe cases, with no major structural brain damage. Patients with more critical injuries only displayed “partial responses” (less than 80% seizure reduction) in 15% of cases.

Bumetanide was used in an open-label clinical trial with promising results. Of the 14 patients treated, 5 had greater than 80% seizure reduction and 2 had greater than 50% seizure reduction after the first dose.22 When combined with phenobarbital, bumetanide resulted in significative seizure reduction in 5 additional patients. Unfortunately, although promising, the trial was stopped early due to ototoxicity concerns; ongoing trials are currently evaluating different treatment protocols and dosing regimens.

A cohort of 76 patients was treated with levetiracetam. As a first line anticonvulsant, used in 22 patients, levetiracetam was effective in providing seizure freedom to 50% of patients after 40 h and to 100% of patients between 100 and 120 h after the initiation of treatment. An overall 92% response rate to levetiracetam as a second-line anticonvulsant after phenobarbital failure was reported in the remaining 54 patients across the 2 studies [46, 47]. When compared with the results obtained in the group treated with phenobarbital first, initial treatment with levetiracetam predicted a shorter interval to seizure freedom in both univariate and multivariate analyses, after adjusting for seizure frequency and HIE severity scores.

### Stroke

Several patients with stroke were reported in different studies. Unfortunately, only data from 45 patients were evaluable for analysis because other patients belonged to larger cohorts that did not stratify results according to etiology (Table 6) [52, 55, 65]. These 45 patients were treated first with phenobarbital, but 43/45 (95.5%) patients required other anticonvulsants, suggesting that phenobarbital may be ineffective for treating stroke-related seizures. In non-responsive patients, 41 were treated with midazolam and lidocaine, as second- or third-line anticonvulsants. In 9 of these patients, the spectral aEEG properties were analyzed, and no data on the clinical efficacy of the administered drugs were available. However, the authors reported that midazolam administration resulted in the moderate suppression of background EEG activity within minutes after the first administration, which lasted for 30–60 min. In contrast, lidocaine administration resulted in a more moderate suppression of background activity and has been reported to suppress electrical activity more strongly within ischemic areas of the brain, suggesting that lidocaine may be more specific and effective for the treatment of specific seizure related etiology [55]. Similar results were reported for the remaining 32 patients with stroke, with lidocaine administration resulting in seizure control in 27/32 patients (84%), compared with the less promising efficacy of midazolam, which was only effective in 5/32 (16%) of the patients treated [52]. Interestingly, the authors reported that the efficacy of lidocaine appeared to be higher in full-term newborns than in preterm newborns and that efficacy appeared to be higher when used as a second-line anticonvulsant after phenytoin, rather than as a third-line AED. One patient with a stroke was reported to have been treated with levetiracetam as a third-line anticonvulsant after phenobarbital and phenytoin failure, with electric remission of seizures 17 min after drug administration [65].

**Table 6:** Treatment outcomes in patients with stroke

Population	First-line AED	Response to first-line AED	Response to Lidocaine as a 2nd−/3rd-line AED	Response to Midazolam as a 2nd−/3rd-line AED	Response to other AEDs
45 newborns with stroke	45 (100%) phenobarbital	4 (5%) did not require further treatment	27 out of 32 (84%) patients treated with lidocaine responded (higher in full-term newborns and when used as a 2nd-line AED)	5 out of 32 (16%) patients treated with midazolam responded	1 patient responded to levetiracetam as a 3rd-line AED

No side effects have been reported associated with the administration of lidocaine or midazolam for stroke patients; however, no long-term follow-up was provided except for the single patient treated with levetiracetam, who was reported to be successfully maintaining seizure-freedom on levetiracetam monotherapy at an 18-month follow-up.

### Genetic Channelopathies

Mutations in genes that encode neuronal ion channels have been associated with a number of early-onset epileptic encephalopathies. A total of 76 patients among case reports, retrospective, and prospective studies were collected in our review (Table 7). In line with the literature, KCNQ2 mutations represented the most common genetic anomalies, associated with early-onset seizures in 86% of the patients included in this review, followed by KCNQ3 mutations (8%) [43, 59–62, 71–76, 85, 86].

**Table 7:** Treatment outcomes of patients with early-onset epileptic encephalopathies

N of patients	Gene mutation	Response to carbamazepine N (%)	Response to lidocaine N (%)	Response to phenytoin N (%)	Response to other AEDS N (%)	Maintenance	Side effects	Follow-up
66	*KCNQ2*	40 (61%) Carbamazepine	4 (6%) lidocaine	7 (10%) phenytoin	13 (20%) combination of drugs, including Na-channel blockers	44 (66%) carbamazepine	1 methemoglobinemia (on lidocaine)	Follow-up: from 3 months to 10 years. Normal development for BFNE; severe developmental delay in *KCNQ2* encephalopathy
6	*KCNQ3*	1 (16%) oxcarbazepine (20 mg/kg)	/	/	2 (33%) levetiracetam (70–85 mg/kg)	1 (16%) oxcarbazepine 2 (33%) levetiracetam	None reported	Normal up to 4 years
1	*KCNT1*	/	/	0 (0%)	No response to phenobarbital, lamotrigine, or benzodiazepines. 1 (100%) levetiracetam 10–30 mg/kg	levetiracetam 30 mg/kg/day	None reported	Seizure decrease (still 1 episode/day) at 14 months
1	*SCN2A*	/	/	1 (100%) phenytoin 20 mg/kg	/	carbamazepine 30 mg/kg	None reported	Severe developmental delay at 2 years
1	*SCN1A*	/	/	/	1 (100%) valproate 50 mg/kg	valproate 50 mg/kg	N/A	Severe developmental delay at 3 years
1	*SCN8A*	0 (0%) oxcarbazepine 80 mg/kg		0 (0%) phenytoin 20 mg/kg	Seizure reduction on a combination of oxcarbazepine, phenytoin, and lamotrigine	phenytoin, oxcarbazepine, phenobarbital, lamotrigine	N/A	Daily seizures at 6 months

Among these 76 patients, 74 were treated with sodium channel blockers, including carbamazepine, phenytoin, lidocaine, and oxcarbazepine, during the courses of their hospital stays. Of these 74 patients, 54 (73%) patients responded to the administration of these drugs, gaining seizure control of the 54 patients that responded to treatment, 41 (76% of patients that responded to treatment) were treated with carbamazepine, [61, 62, 72–74], 8 (15%) were treated with phenytoin, [61, 71] and 4 (7%) were treated with lidocaine [71, 75]. All 12 newborns treated with intravenous phenytoin and/or lidocaine were later transitioned to oral carbamazepine to maintain seizure control. Seizure control was maintained in 46 out of 54 patients (85%) on oral carbamazepine (dose range: 10–30 mg/kg/day).

Based on the seizure type, onset, location, familial anamnesis, and EEG findings, diagnoses of BFNE were made prior to genetic confirmation in 4 patients, who were treated with low-dose oral carbamazepine (10 mg/kg), as a first-line anticonvulsant, and gained seizure freedom within hours of the first administration, with no need for further drug administration [62]. A long-term follow-up of these patients was provided, which demonstrated the maintenance of seizure-freedom for all of them. Among the 20 patients that did not respond, 15 were affected by KCNQ2 epileptic encephalopathy and responded to a combination of several drugs, including sodium channel blockers, topiramate, and levetiracetam [43, 60]; Of the remaining patients, 3 were affected by KCNQ3 encephalopathy, and 2 of these patients dramatically responded to intravenous levetiracetam [43]. Another patient with a KCNT1 mutation was affected by refractory status epilepticus, and seizures decreased with intravenous levetiracetam [67]. One patient had refractory status epilepticus, due to SCN8A mutation [77]; unfortunately, he did not respond to a combination of drugs that included oxcarbazepine, phenobarbital, lamotrigine, and phenytoin.

Among our cohort, 2 patients were never treated with sodium channel blockers; the first was affected by BFNE, responded to phenobarbital, and treatment was slowly tapered until suspension within the first year of age [85]; however, due to the benign course of the condition, seizures may have stopped regardless of the treatment administered. The second patient, in contrast, was diagnosed with SCN1A mutation and was successfully treated with valproate after phenobarbital failure [86].

## Discussion

The therapeutic management of seizures in the newborns has remained unchanged for decades, despite almost 20 years evidence that commonly-used medications are not only ineffective but also potentially neurotoxic for newborns.

This systematic review aimed to collect all of the available data from existing studies published in the literature that have examined the currently available pharmacological treatments of electrically-confirmed neonatal seizures, describing the real-world effectiveness and side-effects associated with drug administration.

Our paper illustrates the limited available evidence regarding the best pharmacological treatments for neonatal seizures and serves as a reference for future studies.

International surveys among neonatologists, worldwide, have confirmed the historical trend toward the use of phenobarbital (in up to 70% of cases), as a first-line AED, and phenytoin (in up to 40% of cases), as a second-line AED, regardless of the seizure etiology or gestational age [90, 91].

However, several studies have demonstrated that phenobarbital may have potential long-term side-effects on neurodevelopment, which is not often considered when making treatment decisions [92–96]. In addition, the overall efficacy of phenobarbital varies widely across reports, and in line with previous data from the literature, our review found that the overall efficacy of phenobarbital does not exceed 66% among all patients treated. Several preclinical studies have explored the poor efficacy of GABAergic drugs, such as phenobarbital and benzodiazepines, by demonstrating that inhibitory mechanisms are underdeveloped in the immature brain, in a manner that is directly proportional to gestational age [97–99]. Animal studies in P7 mice, a post-natal age that grossly corresponds with 30–32 weeks of human gestational age, have confirmed that GABA receptors and the enzymes involved in GABA synthesis are expressed at low levels at birth and increase with time, during the first weeks of life [100]. In particular, the poor efficacy of phenobarbital may represent a developmental consequence of the persistence of the immature form of the sodium-potassium-chloride transporter, NKCC1, which may compromise the chloride-concentration gradient that is essential to phenobarbital’s mechanism of action [101]. Furthermore, GABA is known to act in an excitatory, rather than inhibitory [102], role during early stages of neurodevelopment, which may not only explain the ineffectiveness of GABA-enhancer drugs but also their potential roles during paradoxical seizure disruption.

Phenytoin and lidocaine appear to be potentially effective as second-line treatments for refractory seizures; however, to date, no strong evidence exists to recommend their use.

Phenytoin was shown to be effective in approximately 45% of patients during an RCT [23]. When added as a second-line treatment for seizures that were refractory to phenobarbital, phenytoin facilitated seizure control in an additional 10–15% of treated patients. Different studies have described higher risks of drug accumulation that reach toxic plasma concentrations when administered to preterm compared with full-term newborns. Because of its non-linear pharmacokinetic profile and hepatic metabolism, phenytoin administration also requires frequent blood-level monitoring, making it a slightly manageable medication.

Overall, the effectiveness of lidocaine ranged from 20 to 81% of patients treated [24, 34, 52, 53, 55, 56]; among patients with HIE, however, we observed that only milder phenotypes responded well to lidocaine, whereas a much lower effectiveness rate (30%) was reported for severely asphyxiated newborns [56]. Lidocaine, instead, appears to be more promising for the treatment of patients with stroke [52, 55], and among this population, functional studies demonstrated that lidocaine, in comparison with phenytoin, acted less strongly to suppress background activity and more strongly to suppressing electrical activity in specific ischemic areas of the brain. We observed that lidocaine administration in patients with stroke resulted in seizure control for 84% of patients treated, compared with much lower response rates for both midazolam and phenobarbital. In contrast, several other papers reported potential side-effects associated with lidocaine, including cardiac arrhythmias and hypotension. Therefore, lidocaine should not be used after phenytoin, due to the increased risk of cardio-depressive effects [103]. In addition, a seizure-inducing effect associated with high doses of lidocaine has been reported [104].

In the only RCT that compared midazolam and lidocaine for the treatment of neonatal seizures caused by various etiologies, a toward improved efficacy was observed for lidocaine, although both groups of patients had poor outcomes at 1 year of age [24]. Serious adverse side-effects, such as respiratory depression and sedation, have been reported and potential side-effects may also occur due to interactions between benzodiazepines and other pharmacological treatments. In addition, midazolam clearance correlates with gestational age, with reduced elimination observed among preterm infants, due to immature hepatic metabolism, which may result in a higher risk of side effects due to accumulation [105]. For these reasons, benzodiazepines should be considered second- or third-line treatments that are more suitable for already sedated and intubated newborns.

During the last few years, levetiracetam use has increased, due to the growing amount of literature regarding the safety and efficacy of both loading and maintenance doses and because several studies have reported that levetiracetam, in contrast with phenobarbital, is devoid of any pro-apoptotic properties that might affect the developing brain, even at exceptionally high doses [106, 107]. In addition, both intravenous and enteral preparations are available, making levetiracetam extremely manageable for clinical use. Although the exact mechanism of action for levetiracetam remains unknown, it has been hypothesized to target the synaptic vesicle glycoprotein 2A (SV2A). Talos et al. [108] estimated that neonatal neuronal SV2A protein levels reach 94% of adult values by 37 weeks post-conceptional age, suggesting that the target for levetiracetam may be abundantly expressed, even in the immature neonatal brain.

We have observed that the efficacy of levetiracetam varies across studies, ranging from 32 to 100% of treated patients, for both full-term and preterm newborns [27–30, 38–40, 42, 44–47, 65]. Stratifying patients by etiology allowed us to observe that, to date, more data regarding the efficacy of levetiracetam are available for patients with seizures due to HIE than those due to other causes, and in this population, levetiracetam was effective, providing seizure freedom in up to 50% of patients after 40 h of treatment, when used as a first-line, monotherapy, and in up to 92% of patients in a longer-term follow-up, as both a first- and second-line anticonvulsant [46, 47]. In a population of 44 asphyxiated newborns, initial treatment with levetiracetam predicted a shorter interval to seizure freedom than treatment with phenobarbital in univariate analysis, even after adjusting for initial seizure frequency and unbiased HIE severity score [47]. In addition, comparison between levetiracetam and phenobarbital for the treatment of neonatal seizures caused by various etiologies showed a short-term better effect of levetiracetam on tone and posture of patients according to HNNE score [26]. Treatment doses ranged from 10 to 60 mg/kg for the loading dose, and from 10 to 80 mg/kg for the maintenance dose. No serious adverse events were reported associated with levetiracetam administration, except for mild somnolence and feeding difficulty, which were resolved by dose-adjustment. Several studies focused on the safe and predictable pharmacokinetic profile of levetiracetam, even in preterm and extremely sick full-term newborns, emphasizing that because levetiracetam does not require hepatic metabolism, it rarely interferes with other treatments [109]. Considering its safety profile and higher distribution volume in newborns (0.89 compared with 0.6–0.7 L/kg in children), we recommend the use of higher doses (30–60 mg/kg for the loading dose and 30–50 mg/kg/day, divided into 2–3 doses for maintenance, eventually titrated up to 80 mg/kg/day) [110].

Several papers have described the efficacy of sodium-channel blockers for the treatment of genetic channelopathies. Phenytoin, lidocaine, carbamazepine, and oxcarbazepine act to block the movement of sodium ions through ion channels during the propagation of action potentials to prevent seizure activity. Due to structural similarities, sodium channel blockers also act on potassium channels, resulting in seizure control in patients with genetic epilepsies due to KCNQ mutations. The modulation of one type of channel has also been hypothesized to affect the functions of the entire channel complex.

In agreement with the literature, KCNQ2 mutations represented the most common genetic anomaly identified in our review. We observed a good response to treatment using sodium channel blockers in patients with these mutations, with an overall 63% efficacy. A better response was observed for carbamazepine (77% among responders to treatment), which was also the most commonly used medication because it has few to no reported side-effects and an oral, extremely manageable formulation is available [61, 62, 72–74]. A few patients were treated with lidocaine or phenytoin, who responded to drug administration, were later dismissed on oral carbamazepine for the maintenance of seizure freedom [61, 71, 75, 76].

Interestingly, based on clinical features, familial anamnesis, and EEG patterns, 4 patients were treated early with oral carbamazepine as a first-line anticonvulsant and responded with seizure cessation within hours after the initial first administration [62]. We also observed that the patients who did not respond to carbamazepine were those who displayed features of severe KCNQ2 encephalopathy. Some of these patients responded to combinations of medications that included sodium channel blockers.

Less is known about other genetic encephalopathies, such as the KCNT1-related epilepsy of infancy with migrating focal seizures. Quinidine may effectively block the pathogenic constitutive activation of the KCNT1 channel at the molecular level, but no data regarding its administration for the neonatal population are available, to date [111].

When a genetic channelopathy is suspected, based on clinical features, familial anamnesis, and EEG patterns, the response to treatment with sodium-channel blockers not only represents the best treatment option available but may also be an ex juvantibus criteria to obtain a diagnosis when waiting for genetic test results, encouraging their early use and administration.

A change to the current “one size fits all” treatment model, in which treatment protocols do not account for etiology as a factor, is necessary to accommodate the possibility of customized, patient-specific, precision medicine.

Unfortunately, current data do not yet allow current treatment protocols to be replaced because the populations described, worldwide, are too heterogeneous, both in terms of etiology and treatment. The evaluation of each drug’s efficacy for the treatment of specific etiologies is difficult when the populations described include both preterm and full-term newborns with seizures caused by a variety of etiologies.

Current knowledge, however, allows us to highlight the good clinical and electrographic responses of genetic early-onset epilepsies to sodium channel blockers and the overall good response to levetiracetam, whose administration has also been demonstrated to be safe in both full-term and preterm newborns.

Future investigations should identify methods to better identify and distinguish, as early as possible, between acute seizures and neonatal-onset epilepsies, to facilitate patient-specific, minimally dangerous treatment options, which will offer newborns, especially preterm newborns, higher survival rates, better neurological outcomes, and a better long-term quality of life.

## Conclusions

After more than 20 years of experience, limited evidence exists regarding the best pharmacologic treatments for neonatal seizures. Treatment, too often, remains guided by experience, because few RCTs have been performed and the data available from those that have been performed have not been significant.

Additional controlled trials and large prospective studies are urgently necessary to determine the correct drug choices, dosing regimens, and treatment durations for newborns that will result in better futures, in terms of both seizure freedom and neurocognitive outcome.

This systematic review of neonatal seizure treatment underlines the pitfalls in current neonatology practice and serves as a reference to guide future investigations.

## Data Availability

The datasets used and/or analyzed during the current study are available from the corresponding author on reasonable request.

## Abbreviations

RCT: Randomized clinical trial
EEG: Electroencephalography
aEEG: amplitude-integrated electroencephalography
AED: Antiepileptic drug
WHO: World Health Organization
HIE: Hypoxic-ischemic encephalopathy
IVH: Intraventricular hemorrhage
CNS: Central nervous system
IUGR: Intrauterine growth restriction
BFNE: Benign familial neonatal epilepsy
GABA: Gamma-aminobutyric acid
NKCC1: Sodium-potassium-chloride transporter
SV2A: Synaptic vesicle glycoprotein 2A
HNNE: Hammersmith Neonatal Neurological Examination
